# The Influence of Menstruation and Hormonal Birth Control on the Performance of Female Collegiate Lacrosse Players

**DOI:** 10.3390/sports12110297

**Published:** 2024-10-31

**Authors:** Hannah Humphries, Gabrielle Marchelli, Jennifer A. Bunn

**Affiliations:** 1 College of Osteopathic Medicine, Sam Houston State University, Conroe, TX 77304, USA; hrm047@shsu.edu (H.H.); gam075@shsu.edu (G.M.); 2 College of Health Sciences, Sam Houston State University, Huntsville, TX 77341, USA

**Keywords:** menstrual cycle, female athlete, performance, team sport, contraception

## Abstract

This study compared the mechanical and physiological load placed on Division I female collegiate lacrosse athletes (1) with and without hormone contraceptive (HC) use and (2) with and without menstruation during training and games. Athletes’ (20.6 ± 1.5 years, HC users = 9, naturally cycling (NC) athletes = 9) workloads—total distance traveled (TD, m), max speed (km∙h^−1^), accelerations (repetitions), decelerations (repetitions), and high-intensity distance (HID, m)—were measured through VX Sport wearable microtechnology in training sessions (*n* = 87/athlete) and games (*n* = 17/athlete). Analyses showed no statistical group differences based on HC use or not, and no differences during menstruation versus non-menstruation for training or games. However, while not statistically different, athletes taking HCs performed worse during menstruation, with a 5.1% decline in decelerations, 3.4% decline in TD and HID, 1.2% decline in max speed, and 1% decline in accelerations. NC athletes did not show this same decline with menses. Given that withdrawal bleeding exacerbates performance reduction of HC users, it may be beneficial for these athletes to consider skipping their withdrawal bleed if it is likely to coincide with a game. Further research needs to be carried out to see if these trends are consistent across other female athletes in other sports.

## 1. Introduction

The menstrual cycle (MC) consists of three phases: follicular, ovulatory, and luteal [1,2]. Due to the variability of hormonal changes within two of the phases, they are often further divided into sub-phases: early follicular, late follicular, ovulatory, early luteal, middle luteal, and late luteal. The first 4 to 6 days of the early follicular phase consists of menstruation with low, stable levels of estrogen, progesterone, follicle-stimulating hormone (FSH), and luteinizing hormone (LH) [2]. The late follicular phase begins with a rise of estrogen. When estrogen reaches its peak concentration, there is an increased secretion of gonadotropin-releasing hormone that subsequently results in a spike in LH [2]. This LH spike marks ovulation. The early luteal phase is followed by ovulation, where progesterone is released as a follicle becomes a corpus luteum [1]. To make the endometrium suitable for the implantation of a fertilized egg, there is an influx of progesterone and estrogen. In the late luteal phase, if the egg is not fertilized, the drop in hormones will lead to the shedding of the uterine lining (menstruation), which completes one cycle [1]. For most women, the MC begins with the onset of puberty in the early teen years [1,2,3]. Interruptions to this cycle can include pregnancy, hormonal birth control, and related disorders [4].

The MC affects females both through the hormonal changes that take place over the course of the cycle and the physical act of bleeding [5]. There are many factors that contribute to this, from the subjectivity and variability of symptoms among female athletes, to discomfort many athletes may have discussing their MC with their coaching staff, especially males [6]. At the elite level, male coaches significantly outnumber female coaches [7]. While there are barriers to collecting this data, it will provide beneficial information to improve and better understand female athlete performance [6]. Female collegiate athletes endure the hormonal and physical symptoms associated with menstruation, with the additional requirement of managing rigorous academic workloads on top of the physical demands of training [8,9]. Because there are so many factors influencing female collegiate athlete performance, objective external (mechanical) and internal (physiological) load measurements are combined with subjective wellness assessment scores in order to gain a more complete and holistic picture of an athlete’s performance. These external load measurements have been shown to be significantly affected by an athlete’s sleep quality and energy score on pre-training wellness scores [8]. A higher sleep quality rating predicted an increase in total distance traveled, high-intensity distance traveled, and Athlete Load during practice. Additionally, a higher energy rating predicted an increase in total distance traveled and Athlete Load during practice. This is significant because female athletes often complain of lack of energy, fatigue, and lethargy associated with their MC [10].

The symptoms experienced by female athletes during menstruation include decreased motivation to train, feeling slower and lethargic, physical pain, bloating, headaches, reduced coordination and core strength, uncontrollable emotions, increased worry and stress, easily frustrated, heavy uncontrollable and continual bleeding, reduced confidence, and depression [10]. A recent systematic review has shown that estrogen levels are associated specifically with pain experienced in the temporomandibular joint [11]. In the hope of alleviating and managing these symptoms, 40–50% of female athletes use oral contraceptives (OC) that contain varying levels and types of hormones [12]. There are two oral contraceptive options: (1) the combined pill with estrogen and progestin and (2) the mini pill containing only progestin [13]. OCs are taken daily for three weeks, followed by an “off” week every month. Implants (e.g., Nexplanon, Norplant, and Implanon), hormonal intrauterine devices (IUD, e.g., Mirena and Skyla), and injections (e.g., Dapo-Provera and Noristera) all contain only progestin [13]. Contraceptive patches (e.g., Evra) and vaginal rings (e.g., NuvaRing) release both estrogen and progesterone [12]. While using hormonal contraceptives (HC) may seem advantageous, they cannot guarantee relief from these symptoms and may serve to exacerbate them or cause other side effects in their place [10]. Further, there is not a clear understanding if or how the use of HCs would affect performance.

Choosing to use HC or abstain from them is often a difficult choice for many female athletes due to a lack of clear understanding surrounding how they might impact their athletic performance. Additionally, for those who manage their MCs naturally, previous studies have found that female athletes’ perceived performance tended to be worse in the early follicular and late luteal phases [4]. Perceived performance includes, but is not limited to, reduced motivation, mood disturbances, and lack of concentration [6,10,14]. However, when looking at objective data for natural cycling (NC) athletes, there is a lack of conclusive evidence that any stage of the MC significantly affects physical performance [4]. While there is a lack of evidence for how a specific stage of the MC affects an athlete’s performance, it has been found that HC exacerbates the body’s stress mechanisms in a way similar to that of physical training by increasing circulating levels of cortisol, increasing circulating levels of triglycerides, C-reactive protein (CRP), interleukin-6, and tumor necrosis factor alpha (TNF-α) [5,15]. At every workload, female athletes taking HC experienced higher levels of stress and inflammatory biomarkers than those not taking HC. In response, speculation has been raised regarding the use of HC in combination with high-demand physical training as it alters biomarkers—indicating exacerbated stress and inflammatory responses—in female athletes that may require an increased need for recovery in these athletes. States of increased systemic inflammation cannot only affect an athlete’s ability to adapt to training but also increase an athlete’s risk for cardiovascular disease due to an elevated CRP [16]. Increased fat mass and body weight reported by HC-using athletes may affect performance outcomes; however, current research outcomes are variable [5,17,18,19]. Furthermore, it is unknown if an athlete who uses an HC experiences any negative effects related to a withdrawal bleed (placebo week) or if there are negative effects associated with avoiding a withdrawal bleed.

Research on how the MC and its associated symptoms affect female athletes’ ability to perform has been significantly underrepresented in the literature over the years [20,21]. Sports science research has been male-centered due to the perceived unpredictability of the female menstrual cycle and the long tradition of sports being dominated by males until the late 1800s [20,21,22,23,24,25]. The aim of this study was to compare the mechanical and physiological load (total distance traveled (TD), maximum speed, acceleration, deceleration, and high-intensity distance traveled (HID)) placed on Division I female collegiate lacrosse athletes (1) with and without HC use and (2) with and without menstruation during training sessions and game matches. We hypothesized that athletes would experience reduced performance during their menses or withdrawal bleeds compared to when they were not menstruating. We also hypothesized that there would be no differences in performance between those using HCs and those without.

## 2. Materials and Methods

### 2.1. Experimental Approach to the Problem

This observational study investigated the objective mechanical and physiological load experienced by female athletes during the competitive lacrosse season over 15 weeks. The independent variables included whether the athletes were menstruating or not and whether the athletes used an HC. The dependent variables included on-field workload variables measured through microtechnology during games and training. This study was conducted in accordance with and approved by the university Institutional Review Board (CU-IRB 705). Informed consent was obtained from all participants, and they were allowed to ask questions regarding the study before data collection. All participants confirmed that they understood the study, including the associated benefits and risks. Data collection took place during the 2024 Division I competitive season.

### 2.2. Participants

A priori analysis using G*Power for 80% power and an alpha level of 0.05 indicated that a total of 16 athletes needed to be enrolled, with eight per group [5,26]. Analysis used both energy expenditure and PlayerLoad in the calculations to determine the sample size. Division I female lacrosse players (n = 26) competing on the same team were enrolled in this study. Female athletes on the varsity lacrosse team who were aged 18 or older at the time of this study and issued a global positioning system (GPS) tracking unit were eligible to be included. Additionally, eligible subjects were required to be cleared for play by a licensed athletic trainer and team physician. Grounds for exclusion included individuals removed from the team or chose to withdraw from the team, injured individuals who missed 30% or more of the practices and games (n = 3), and athletes taking OC who continued to take their OC pills during the withdrawal week to avoid menstruation (n = 5). The final analysis included data from 18 team members (20.6 ± 1.5 years, 166 ± 8 cm). Athletes trained and competed according to their team schedule and in compliance with the National Collegiate Athletics Association’s rules and regulations.

### 2.3. Instrumentation and Data Collection

Objective mechanical and physiological loads were collected each day of training and games. A total of 1855 individual data files were collected—1549 training session data files (average of 87/athlete) and 306 gameday session files (17/athlete).

The external load was measured through wearable microtechnology (VX Sport, Wellington, New Zealand). VX Sport units included a GPS unit (collecting at 10 Hz) and heart rate monitor (collecting at 2.4 GHz) that were deemed to obtain reliable internal and external load measurements [27,28]. Each athlete was given their own vest and device for the duration of the data collection period, in which the microtechnology units were placed. The GPS units obtained satellite connection per manufacturer recommendations before a game or practice session and then placed on the thoracic spine between the shoulder blades. After each training session and game, the GPS data were uploaded to the VX Sport Training Tool software program and then trimmed to remove inactive periods and link the drills and activities completed to that day’s data. Data were sorted in accordance with the coaches’ daily practice plan. The metrics used for external training load included: TD moved in meters, maximum speed (km∙h^−1^), accelerations (count, >3 m∙s^−1^), decelerations (count, >3 m∙s^−1^), and HID (meters completed > 60% maximum sprint speed). Maximum sprint speed was obtained for each athlete by using the fastest time they achieved from three 50-m sprint tests, with a 20-m fly-in, followed by a 30-m “all-out” sprint effort [27]. Variables were selected to align with previous lacrosse literature [29,30,31].

Athletes completed a shortened menstrual cycle status questionnaire to indicate their use of HCs or if they were NC. Athletes using HCs indicated the type of HC used, and athletes who were NC shared the length of their MC, length of menses, and regularity of their MC. This survey aligns with similar tools used in previous research [5]. To track menstruation, athletes indicated whether they were menstruating or not on their daily wellness platform (Metrifit, Louth, Ireland). For HC users, they indicated if they were having a withdrawal bleed or not. Metrifit tracked the length of each athlete’s cycle and the average number of days of menstruation. Athletes completed these questions daily throughout the duration of the competitive season.

### 2.4. Statistical Analysis

Athlete data was separated into training and gamedays. The average of each dependent variable was calculated for each athlete during menstruation and non-menstruation for training and games. Means and standard deviations for each group—HC or NC—were calculated. The difference and percent change between menstruation conditions for each group was also calculated. Data were determined to be normally distributed via a Shapiro-Wilks test. Two (one for training data and one for gameday data) factorial analyses of variance (ANOVA) were used to evaluate group (HC vs. NC) and menstruation differences (menses vs non-menses) in performance. Partial eta-squared effect sizes (ES) were used to determine the magnitude of the differences and were interpreted as small (0.01), moderate (0.06), and large (0.14) [32]. SPSS (version 27.0, Chicago, IL, USA) was used for all analyses, and an alpha level of 0.05 was used to determine differences.

## 3. Results

Data were analyzed for 18 individuals, 9 using HC (8 athletes used an OC and 1 used an IUD) and 9 NC (56% classified as eumenorrheic). The average number of days for a total pill cycle was 26.9 ± 1.9 days for HC users, and the average length of the menstrual cycle was 31.5 ± 3.6 days for the NC athletes. The mean length of menses was 5.6 ± 1.6 days for HC users and 6.3 ± 2.5 for NC athletes. The number of cycles tracked over the four-month competitive season was 2.8 ± 1.3 for HC users and 2.6 ± 1.1 for NC athletes.

### 3.1. Games

The factorial ANOVA showed no main effect differences for HC use (λ (5, 12) = 0.546, *p* = 0.739, ES = 0.185, large), menses versus non-menses (λ (5, 12) = 0.840, *p* = 0.547, ES = 0.259, large), or an interaction between the two (λ (5, 12) = 0.153, *p* = 0.975, ES = 0.060, moderate). Table 1 shows the means and standard deviations for HC and NC groups for gameday performance measures. An athlete’s menstruation status or use of HCs did not statistically affect their game performance. Calculations showing the difference and percent change in non-menstruating from menstruating status per group are shown as “difference” in Table 1. Only the HC group had a consistent, albeit not statistically different, reduction in-game performance by 1–5% when they experienced their withdrawal bleed. The NC group showed a menses-related reduction of 1.5% for only maximum speed. All differences are within one standard deviation of each mean.

### 3.2. Training

Table 2 shows the means and standard deviations for HC and NC groups for training performance measures. The RM-ANOVA showed no main effect differences for HC use (λ (5, 14) = 1.014, *p* = 0.446, ES = 0.266, large), menses versus non-menses (λ (5, 14) = 0.585, *p* = 0.712, ES = 0.173, large), or an interaction between the two (λ (5, 14) = 1.984, *p* = 0.144, ES = 0.415, large). Similar to the gameday data, an athlete’s menstruation status or HC use did not statistically affect their performance during training sessions. There were fewer reductions in performance during training for the HC group during their withdrawal bleed, with only TD showing a 1.2% reduction. However, the HC group increased HID by 18% during their withdrawal bleed. For the NC group, there was an 8.4% reduction during menses in HID. Again, all differences are within one standard deviation of their respective mean.

## 4. Discussion

This study evaluated the athletic performance of female athletes and whether there were differences in performance during menses and with the use of HCs. Athletic performance was measured by the mechanical and physiological load placed on these athletes during games and training sessions. No statistical differences were found in training or games between those using HCs and those not using HCs or between those menstruating and those not menstruating. However, notable declines in game performance of the HC group during their withdrawal bleed were shown. While these differences were not statistically different, they are likely impactful in high-level game performance [33].

There were no statistical differences in external load between athletes who were menstruating and those who were not menstruating during training or gamedays. This result is consistent with previous research on objective performance in menstruating athletes [4,34]. While many female athletes believe their objective performance is affected by menstruation, most studies show no change in performance across any phase of the MC [34]. For example, in a study looking at female running speed in hot conditions, there were no differences in maximal 15-m sprint effort or the Loughborough Intermittent Shuttle Test [35]. Additionally, elite-level Tunisian female soccer players showed no difference in sprint ability or max jump height according to the MC phase [36]. However, previous literature has found that the worst performance experienced by athletes was reported during menstruation, and the best performance was reported following menstruation [37,38]. The previous literature was conducted via exercise tests and failed to study on-field/court athlete performance objectively through measurable data like TD, maximum speed, acceleration, deceleration, HID. The present study is expressly different from these previous trials because it was conducted in an applied setting. Notably, the previous literature related to exercise performance using controlled lab tests, may not always translate to practical and ecological training and game scenarios.

Similar to measuring clinical change, the difference between a successful or unsuccessful performance may not always be statistically significant at the highest level of athletic performance. When conducting research on the optimal performance of elite athletes, reliance on the common measure of statistical significance could be a limitation in determining meaningful effects for an athlete’s performance [4]. This was seen in the women’s 100 m and 200 m freestyle swimming events in the 1992 Olympics. While no statistical significance was found between the early follicular and late luteal phase, swimmers in the late luteal group swam 2.3 s faster in the 100 m and 7.3 s faster in the 200 m than those in the early follicular group. This is notable because 1.95 s separated the 1st and 8th places [4]. In the present study, there was a decrease in gameday performance across the board for the players who took HC and were menstruating, ranging from 1.2% to 5.1% reduction in performance. HC users performed worse in all objective measures during the game, whereas the NC group only showed a 0.4% reduction in maximum speed. Previous lacrosse literature has shown performance declines through a game’s progression [33,39,40]. The performance reduction of HC users during a game appears to be exacerbated by a withdrawal bleed. Similarly, Bozzini et al. showed consistently reduced performance in HC users compared to their NC counterparts in collegiate soccer athletes [5]. Bozzini did not consider time points of menstruation, but the athletes showed weekly reductions in PlayerLoad and energy expenditure [5]. The results of the present study suggest that it may be prudent for HC users to consider skipping their withdrawal bleed if it is likely to coincide with a game.

Further research needs to be carried out to see if these trends continue to be seen in female athletes in other sports. Athletes and coaches should consider individual analyses to see if HC use is a detriment to the athlete. Considerations of perceived performance and menstrual-related symptoms should also be included in any decision-making.

This study contained some limitations. First, accurate staging of where an athlete was in their MC is unobtainable without regular hormonal blood sampling and/or urinary ovulation markers [41]. Thus, we are unable to make assumptions about menstrual phases with the athletes while they were outside of their menses, and the data were collected via self-report. Second, this study lacked variation in HC options that are used by athletes (8 athletes used an OC, and 1 used an IUD). The direct effects of different types of HC on athletic performance are not fully known and need further investigation. Third, the study only looked at one collegiate lacrosse team; therefore, the obtained data cannot be generally applied to female athletes at this time. Lastly, the study included calculated differences and percent change, whereas adjustments made based on play time may be useful in future studies. Some strengths of the study include practical on-field training and game data with a collegiate team during their competitive season. The variations in HC use and variations in the eumenorrheic status of NC athletes are real-world issues that many female athletes and teams experience. Managing these scenarios for individual athletes and in conjunction with a team sport is relevant for coaches and support staff to understand. The present study also only analyzed differences in the MC that the athlete physically sees and knows.

This study provides female athletes with a sense of freedom and empowerment, knowing that whether or not they are menstruating or undergoing withdrawal bleeding, their ability to perform does not appear to be significantly affected. Athletes do not need to fear that their menstruation hinders their objective, athletic performance. Further research should be undertaken on elite female athlete performance to see if smaller percentage changes in objective measures affect successful outcomes in team sports across the board.

## 5. Conclusions

The results showed no statistical differences in game or training performance between NC athletes and athletes using HCs or with the effect of menstruation/withdrawal bleed. Upon further inspection, the data showed small reductions in the gameday performance of HC users during their withdrawal bleed. While not statistically different, these reductions may serve as key differences in game performance that could alter the result of a game. Athletes who experience these reductions should consider forgoing their withdrawal bleed and evaluate if there are any differences. Coaches should work to create an environment that is open for communication on this topic, especially as it relates to an athlete’s performance and wellbeing. As studies continue to be carried out in this area, there appears to be a discrepancy between athletes’ perceived performance and their objective performance [4]. While these data reveal that neither HC nor menstruation affected a female athlete’s objective performance, perceived performance measures for these athletes reveal that menstruation does affect an athlete’s psychology [4,38]. Future research should be conducted to intervene (e.g., cognitive behavior therapy) or treat (e.g., medication) this misperception so that these athletes can perform physically and psychologically at their highest level. Understanding the reason for this discrepancy seems more beneficial, such that, in understanding the mental plight of these female athletes, there may be a way to intervene to relieve them and help their perception catch up to the reality of their physicality.

## Figures and Tables

**Table 1:** External output data on gameday for lacrosse players with and without menstruation and with and without HC use. The difference and percent change (shown in parentheses) between menses and not menses are shown for each variable. The down arrow indicates performance during menses or withdrawal bleed was lower than non-menses.

Variable	NC Group	HC Group
TD (m)		
Menses	6147 ± 2059	6791 ± 2865
Not menses	6010 ± 2165	7030 ± 2307
Difference	136 (2.2%)	↓240 (3.4%)
Max speed (km/h)		
Menses	24.7 ± 2.3	24.6 ± 2.7
Not menses	25.0 ± 1.3	24.9 ± 2.3
Difference	↓0.4 (1.5%)	↓0.30 (1.2%)
Accelerations (reps)		
Menses	106.5 ± 44.8	113.8 ± 61.3
Not menses	99.5 ± 45.5	114.9 ± 53.6
Difference	7 (7%)	↓1 (1%)
Decelerations (reps)		
Menses	27.5 ± 15.9	31.3 ± 18.1
Not menses	27.1 ± 15.2	31.3 ± 18.1
Difference	0.4 (1.4%)	↓1.6 (5.1%)
HID (m)		
Menses	520.0 ± 366.3	548.6 ± 391.3
Not menses	487.0 ± 395.5	568.0 ± 332.5
Difference	33 (6.8%)	↓19 (3.4%)

Note: normal cycling (NC), hormone contraceptive (HC), total distance (TD), and high-intensity distance (HID), ↓ indicates a reduction in performance during menses compared to non-menses.

**Table 2:** External output data in training sessions for lacrosse players with and without menstruation and with and without HC use. The difference and percent change (shown in parentheses) between menses and not menses are shown for each variable. The down arrow indicates performance during menses or withdrawal bleed was lower than non-menses.

Variable	NC Group	HC Group
TD (m)		
Menses	4733 ± 736	4378 ± 732
Not menses	4558 ± 559	4430 ± 594
Difference	175 (3.7%)	↓52 (1.2%)
Max speed (km/h)		
Menses	24.0 ± 1.6	23.7 ± 1.6
Not menses	23.8 ± 1.2	23.4 ± 1.2
Difference	0.2 (0.8%)	0.3 (1.1%)
Accelerations (reps)		
Menses	93.0 ± 20.4	84.1 ± 24.8
Not menses	91.6 ± 20.4	83.8 ± 21.0
Difference	1.4 (1.5%)	0.3 (0.4%)
Decelerations (reps)		
Menses	25.9 ± 8.1	23.8 ± 20.3
Not menses	24.4 ± 7.8	23.2 ± 8.8
Difference	1.5 (6.0%)	0.6 (2.9%)
HID (m)		
Menses	308.1 ± 173.4	367.5 ± 240.9
Not menses	336.4 ± 108.5	311.1 ± 90.2
Difference	↓28.3 (8.4%)	56.4 (18.1%)

Note: normal cycling (NC), hormone contraceptive (HC), total distance (TD), and high-intensity distance (HID), ↓ indicates a reduction in performance during menses compared to non-menses.

## Data Availability

Data can be found at Bunn, Jennifer (2024), “Lacrosse—wellness, performance, and menstrual cycle”, Mendeley Data, V2, https://data.mendeley.com/datasets/c2sw26g6cp/1.

## References

[1] Holesh J.E., Bass A.N., Lord M. (2024). Physiology, Ovulation. StatPearls.

[2] Mihm M., Gangooly S., Muttukrishna S. (2011). The Normal Menstrual Cycle in Women. Anim. Reprod. Sci..

[3] Reed B.G., Carr B.R., Feingold K.R., Anawalt B., Blackman M.R., Boyce A., Chrousos G., Corpas E., de Herder W.W., Dhatariya K. (2000). The Normal Menstrual Cycle and the Control of Ovulation. Endotext.

[4] Carmichael M.A., Thomson R.L., Moran L.J., Wycherley T.P. (2021). The Impact of Menstrual Cycle Phase on Athletes’ Performance: A Narrative Review. Int. J. Env. Res. Public Health.

[5] Bozzini B.N., McFadden B.A., Elliott-Sale K.J., Swinton P.A., Arent S.M. (2021). Evaluating the Effects of Oral Contraceptive Use on Biomarkers and Body Composition during a Competitive Season in Collegiate Female Soccer Players. J. Appl. Physiol..

[6] Findlay R.J., Macrae E.H.R., Whyte I.Y., Easton C., Forrest (Née Whyte) L.J. (2020). How the Menstrual Cycle and Menstruation Affect Sporting Performance: Experiences and Perceptions of Elite Female Rugby Players. Br. J. Sports Med..

[7] De Haan D., Sotiriadou P. (2019). An Analysis of the Multi-Level Factors Affecting the Coaching of Elite Women Athletes. Manag. Sport Leis..

[8] Crouch A.K., Jiroutek M.R., Snarr R.L., Bunn J.A. (2021). Relationship between Pre-Training Wellness Scores and Internal and External Training Loads in a Division I Women’s Lacrosse Team. J. Sports Sci..

[9] Favero T.G., White J. (2018). Periodization in College Soccer. Strength Cond. J..

[10] Brown N., Knight C.J., Forrest (Née Whyte) L.J. (2021). Elite Female Athletes’ Experiences and Perceptions of the Menstrual Cycle on Training and Sport Performance. Scand. Med. Sci. Sports.

[11] Zieliński G., Pająk-Zielińska B. (2024). Association between Estrogen Levels and Temporomandibular Disorders: An Updated Systematic Review. Int. J. Mol. Sci..

[12] Martin D., Elliott-Sale K., Nottingham Trent University, United Kingdom (2016). A Perspective on Current Research Investigating the Effects of Hormonal Contraceptives on Determinants of Female Athlete Performance. Rev. Bras. Educ. Fís. Esporte.

[13] Powell A. (2017). Choosing the Right Oral Contraceptive Pill for Teens. Pediatr. Clin. N. Am..

[14] O’Brien S., Rapkin A., Dennerstein L., Nevatte T. (2011). Diagnosis and Management of Premenstrual Disorders. BMJ.

[15] Hertel J., König J., Homuth G., Van Der Auwera S., Wittfeld K., Pietzner M., Kacprowski T., Pfeiffer L., Kretschmer A., Waldenberger M. (2017). Evidence for Stress-like Alterations in the HPA-Axis in Women Taking Oral Contraceptives. Sci. Rep..

[16] Pepys M.B., Hirschfield G.M. (2003). C-Reactive Protein: A Critical Update. J. Clin. Investig..

[17] Casazza G.A., Suh S.-H., Miller B.F., Navazio F.M., Brooks G.A. (2002). Effects of Oral Contraceptives on Peak Exercise Capacity. J. Appl. Physiol..

[18] Procter-Gray E., Cobb K.L., Crawford S.L., Bachrach L.K., Chirra A., Sowers M., Greendale G.A., Nieves J.W., Kent K., Kelsey J.L. (2008). Effect of Oral Contraceptives on Weight and Body Composition in Young Female Runners. Med. Sci. Sports Exerc..

[19] Rickenlund A., Carlström K., Ekblom B., Brismar T.B., Von Schoultz B., Hirschberg A.L. (2004). Effects of Oral Contraceptives on Body Composition and Physical Performance in Female Athletes. J. Clin. Endocrinol. Metab..

[20] Costello J.T., Bieuzen F., Bleakley C.M. (2014). Where Are All the Female Participants in Sports and Exercise Medicine Research?. Eur. J. Sport Sci..

[21] Cowley E.S., Olenick A.A., McNulty K.L., Ross E.Z. (2021). “Invisible Sportswomen”: The Sex Data Gap in Sport and Exercise Science Research. Women Sport Phys. Act. J..

[22] McNulty K., Olenick A., Moore S., Cowley E. (2024). Invisibility of Female Participants in Midlife and beyond in Sport and Exercise Science Research: A Call to Action. Br. J. Sports Med..

[23] Anderson N., Robinson D.G., Verhagen E., Fagher K., Edouard P., Rojas-Valverde D., Ahmed O.H., Jederström M., Usacka L., Benoit-Piau J. (2023). Under-Representation of Women Is Alive and Well in Sport and Exercise Medicine: What It Looks like and What We Can Do about It. BMJ Open Sport Exerc. Med..

[24] Fagher K., Verhagen E. (2022). When Women Can Be Stars in Sports, Why Is It so Difficult in Sports and Exercise Medicine Research?. BMJ Open Sport Exerc. Med..

[25] Cowan S.M., Kemp J.L., Ardern C.L., Thornton J.S., Rio E.K., Bruder A.M., Mosler A.B., Patterson B., Haberfield M., Roughead E.A. (2023). Sport and Exercise Medicine/Physiotherapy Publishing Has a Gender/Sex Equity Problem: We Need Action Now!. Br. J. Sports Med..

[26] Kang H. (2021). Sample Size Determination and Power Analysis Using the G*Power Software. J. Educ. Eval. Health Prof..

[27] Alphin K.L., Sisson O.M., Hudgins B.L., Bunn J.A. (2020). Accuracy Assessment of a GPS Device for Maximum Sprint Speed. Int. J. Exerc. Sci..

[28] Malone S., Doran D., Collins K., Morton J., McRoberts A. Accuracy and Reliability of the VXSport Global Positioning System in Intermittent Activity. Proceedings of the 19th Annual Congress of the European College of Sport Science.

[29] Bunn J.A. (2022). An Evaluation of Internal and External Workload Metrics in Games in Women’s Collegiate Lacrosse. J. Sport Exerc. Sci..

[30] Bunn J.A., Myers B.J., Reagor M.K. (2021). An Evaluation of Training Load Measures for Drills in Women’s Collegiate Lacrosse. Int. J. Sports Physiol. Perform..

[31] Bynum L., Snarr R.L., Myers B.J., Bunn J.A. (2022). Assessment of Relationships Between External Load Metrics and Game Performance in Women’s Lacrosse. Int. J. Exerc. Sci.

[32] Cohen J. (1988). Statistical Power Analysis for the Behavioral Sciences.

[33] Thornton A.R., Figueroa Y., Davis P., Bunn J.A. (2023). Comparision of Game Data Between Halves and Quarters in Division I Women’s Lacrosse. J. Sci. Sport Exerc..

[34] Vogel K., Larsen B., McLellan C., Bird S.P. (2023). Female Athletes and the Menstrual Cycle in Team Sports: Current State of Play and Considerations for Future Research. Sports.

[35] Sunderland C., Nevill M. (2003). Effect of the Menstrual Cycle on Performance of Intermittent, High-Intensity Shuttle Running in a Hot Environment. Eur. J. Appl. Physiol..

[36] Tounsi M., Jaafar H., Aloui A., Souissi N. (2018). Soccer-Related Performance in Eumenorrheic Tunisian High-Level Soccer Players: Effects of Menstrual Cycle Phase and Moment of Day. J. Sports Med. Phys. Fit..

[37] Jacobson B.H., Lentz W. (1998). Perception of Physical Variables during Four Phases of the Menstrual Cycle. Percept. Mot Skills.

[38] Solli G.S., Sandbakk S.B., Noordhof D.A., Ihalainen J.K., Sandbakk Ø. (2020). Changes in Self-Reported Physical Fitness, Performance, and Side Effects Across the Phases of the Menstrual Cycle Among Competitive Endurance Athletes. Int. J. Sports Physiol. Perform..

[39] Calder A.R., Duthie G.M., Johnston R.D., Engel H.D. (2021). Physical Demands of Female Collegiate Lacrosse Competition: Whole-Match and Peak Periods Analysis. Sport Sci. Health.

[40] Kilian J., Snyman K., Miyashita T. (2022). Comparison of In-Game External Load Metrics Among Positions and between Halves for Division I Collegiate Women’s Lacrosse Athletes. Int. J. Strength Cond..

[41] Colenso-Semple L.M., D’Souza A.C., Elliott-Sale K.J., Phillips S.M. (2023). Current Evidence Shows No Influence of Women’s Menstrual Cycle Phase on Acute Strength Performance or Adaptations to Resistance Exercise Training. Front. Sports Act. Living.

